# Dr. B. C. Bapna: A tough task master and a crusader

**DOI:** 10.4103/0970-1591.60435

**Published:** 2010

**Authors:** S. K. Sharma

**Affiliations:** Department of Urology and Director, PGIMER, Chandigarh - 160 012, India. E-mail: skssilveroaks@gmail.com


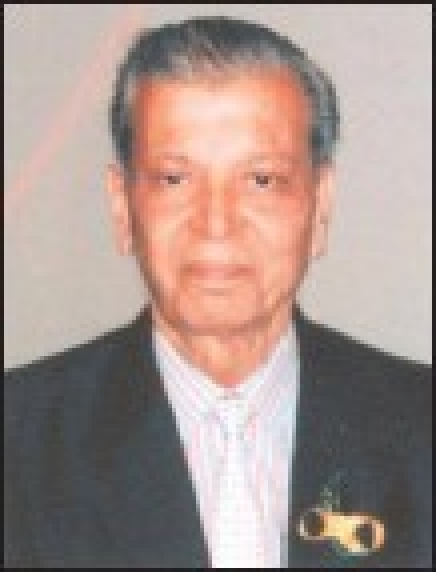


One of the founding fathers of Indian Urology, Professor B. C. Bapna, was born on 1^st^ July 1927 at Indore. After his initial schooling and intermediate at Indore, he joined Medical College, Nagpur, and completed his M.B.B.S in 1952. For the next 2–3 years, he served as a House Surgeon and a Registrar at Medical College, Indore. In 1955, in pursuit of higher specialized education, he moved to the USA. He completed his 4-year residency in Urology at Kansas City Hospital, Kansas, and cleared FRCS in Urology at the Royal Canadian College of Surgeons. He decided to return to his motherland in the hope of initiating Urology, which hardly existed as a speciality then.

On his return to India in 1961, he visited few places, but in the absence of a suitable opportunity and his ardent love for Urology, he joined as a Registrar in the Department of General Surgery at the All India Institute of Medical Sciences (AIIMS), New Delhi. He started performing endoscopic urological procedures with his own set of ACMI instruments, which he had brought from the USA. This made him very popular at AIIMS. The speciality of Urology started at AIIMS in 1963 with Late Prof. S. M. Singh as Associate Professor and Dr. B. C. Bapna as Assistant Professor. In 1969, he was promoted as Associate Professor of Urology and headed a separate unit.

In 1971, after the sudden demise of Late P. N. Katawa, Dr. Bapna was selected as Professor and Head of Urology at the Postgraduate Institute of Medical Education and Research, Chandigarh (PGIMER). He was joined by Dr. M. S. Rao as Assistant Professor in 1972 and myself as a Lecturer in 1975. Dr. S. Vardyanathan, who passed his M.Ch under Professor Bapna, became a Lecture later on.

Prof. Bapna was a strict disciplinarian; a hard task master and brooked no non-sense. A reprimand to the defaulting Senior Resident and even to the faculty members would come in the choicest words that would put them in place. He trained scores of specialists, both at AIIMS and at PGIMER, who occupied coveted and places of pride not only in India but in the other countries too. Prof. Bapna, besides being a great teacher, was also a master craftsman as an endoscopic surgeon. Before the advent of the fiber optic light sources, he practiced his art with battery-cell-operated lamps. It was always a pleasure to watch him operate.

He established probably one of the best centers of Urology training and research at PGI, Chandigarh, that it became the envy of the best brains in the country, who longed to get admission for M.Ch Urology. Under his leadership, a number of research projects under the aegis of the Indian Council of Medical Research (ICMR), Department of Science and Technology (DST) and Indian National Science Academy (INSA) and a generous funding from the Institute were initiated. These encompassed the endemic renal stone disease in this region: national highway trauma and neurogenic bladder and complicated vesico-vaginal fistulae as a consequence of neglected labour in poor rural areas. All these efforts resulted in the publication of a very large number of articles in reputed and peer-reviewed national and international journals. The center at PGI came on the world map of Urology.

The speciality of urology started in India in 1960–1 as a section of Urology in the larger ambit of the Association of Surgeon of India. Dr. Bapna played a stellar role in the formative years as its Secretary- cum-Treasurer for a long time. He gave it a definite direction as well as monitored its growth. He was elected as President of the Urological Society of India for a 2-year term in 1979–80. Prof. Bapna also became a fellow of the National Academy of Medical Sciences in India.

He superannuated in June 1986 and moved to Indore a year later. He practiced for some time in the beginning only. At the age of 83 years, Prof. Bapna continues to be in the pink of health and has a wonderful time in the company of his children and grandchildren. I have probably had the longest association with Prof. Bapna, first as his student and later on as his colleague. It was indeed a great pleasure and a challenge. I learnt a lot from him, which enabled me to wade through comfortably in troubled situations. I wish Mrs. and Prof. Bapna many more years of a healthy and peaceful life.

